# Methylene Blue in Septic Shock: Emerging Evidence, Clinical Applications, and Future Directions

**DOI:** 10.7759/cureus.86701

**Published:** 2025-06-24

**Authors:** Namrata Maheshwari, Bipin Malkania, Rasil Mudiyanselage

**Affiliations:** 1 Critical Care Medicine, Medway Maritime Hospital, Gillingham, GBR; 2 Intensive Care Unit, Medway NHS Foundation Trust, Gillingham, GBR

**Keywords:** catecholamine-sparing therapy, cyclic gmp pathway, methylene blue, nitric oxide inhibition, refractory septic shock, vasopressin comparison, vasopressor requirements

## Abstract

Septic shock remains a significant cause of mortality in intensive care units worldwide despite advances in management strategies. The pathophysiology involves profound circulatory and cellular abnormalities leading to vasoplegic shock. While norepinephrine remains the first-line vasopressor, there is growing interest in catecholamine-sparing agents to mitigate the adverse effects of adrenergic overstimulation. Methylene blue, with its inhibitory action on nitric oxide pathways, has emerged as a potential adjunctive therapy. This review examines the theoretical mechanisms, pharmacokinetics, clinical evidence, and practical considerations for methylene blue use in septic shock. While some promising results regarding reduced vasopressor requirements and length of stay are suggested, current evidence does not support methylene blue as a replacement for established second-line agents like vasopressin. Further large-scale randomized trials are needed to establish optimal dosing regimens, timing of administration, and specific patient populations who might benefit most from this intervention.

## Introduction and background

Septic shock, defined by the Sepsis-3 consensus as "a subset of sepsis in which underlying circulatory and cellular/metabolic abnormalities are profound enough to substantially increase mortality" [1], represents the most severe manifestation of the host response to infection, with mortality rates approaching 40%-60% in some series [2,3]. Despite advances in critical care, the complex pathophysiology of septic shock-involving vasodilation, impaired myocardial function, microcirculatory dysfunction, and cellular metabolic derangements-continues to challenge clinicians [4].

Current management strategies center on fluid resuscitation and vasopressor therapy, with norepinephrine as the first-line agent due to its predominantly α-1 adrenergic effects causing vasoconstriction and limited β-1 activity supporting cardiac output [5,6]. However, concerns about the adverse effects of prolonged or high-dose catecholamine therapy, including myocardial ischemia, arrhythmias, digital ischemia, hyperglycemia, immunosuppression, and increased mortality [7,8], have driven interest in catecholamine-sparing strategies.

Current guidelines recommend vasopressin as a second-line agent in septic shock refractory to norepinephrine [9]. Other agents include angiotensin II [10] and corticosteroids [11]. Methylene blue, originally discovered in the 19th century and known for various medical applications including the treatment of methemoglobinemia and as a surgical dye, has gained renewed attention for its potential role in managing vasoplegic shock due to its mechanism of action directly targeting the nitric oxide (NO) pathway, which plays a central role in the pathophysiology of septic vasodilation [12].

## Review

Methylene blue: pharmacology and mechanism of action

Methylene blue (methylthioninium chloride) is an aromatic heterocyclic compound with the chemical formula C_16_H_18_ClN_3_S and a molecular weight of 319.85 g/mol [13] that appears as a dark green crystalline powder dissolving in water to produce a characteristic deep blue solution. In clinical settings, it is available as a 1% (10 mg/mL) solution for intravenous administration [14].

Following intravenous administration, methylene blue demonstrates complex pharmacokinetics with a tri-exponential decline in plasma concentration, having a large volume of distribution (255 ± 58 L) that indicates extensive tissue uptake, and is approximately 94% protein-bound [15,16]. Peak plasma concentrations are achieved within 30 minutes of administration, with clinical effects typically observed 30-60 minutes after infusion begins [17]. The elimination half-life is approximately 5.25 hours in healthy individuals, though this may be prolonged in patients with renal or hepatic dysfunction [18]. Methylene blue is metabolized in the tissues by NADPH (nicotinamide adenine dinucleotide phosphate) reductases to leucomethylene blue (colorless reduced form), which is predominantly excreted in urine and gives it a characteristic blue-green color, with approximately 75% of the administered dose excreted in urine within 12 hours, and a small portion eliminated unchanged [19].

The pathophysiology of vasodilation in septic shock involves excessive production of NO, a potent vasodilator synthesized from L-arginine by NO synthase (NOS) enzymes. There are three main isoforms of NOS: endothelial NOS (eNOS), constitutively expressed in endothelial cells, producing low levels of NO for physiological vasodilation; neuronal NOS (nNOS), constitutively expressed in neuronal tissue; and inducible NOS (iNOS), expressed in response to inflammatory stimuli, particularly endotoxins and cytokines including IL-1, IL-6, TNF-α, and IFN-γ [20].

Both constitutive forms (eNOS and nNOS) are calcium-dependent and regulated through negative feedback mechanisms, while iNOS is calcium-independent and, once induced, produces large quantities of NO for prolonged periods without feedback regulation [21]. During septic shock, the inflammatory cascade triggers massive iNOS upregulation, leading to excessive NO production. NO exerts its vasodilatory effect by activating soluble guanylate cyclase (sGC), which catalyzes the conversion of guanosine triphosphate (GTP) to cyclic guanosine monophosphate (cGMP). Increased intracellular cGMP activates protein kinase G, leading to reduced intracellular calcium through sarco/endoplasmic reticulum calcium ATPase (SERCA) activation, myosin light chain phosphatase activation causing myosin dephosphorylation, and hyperpolarization through the activation of potassium channels [22,23], with these combined actions resulting in vascular smooth muscle relaxation and vasodilation.

Methylene blue interrupts this pathway through two primary mechanisms: direct inhibition of iNOS, limiting NO production at its source [24], and inhibition of sGC by binding to the iron heme moiety of sGC, which prevents NO-induced activation of the enzyme, thereby reducing cGMP formation and its downstream vasodilatory effects [25,26]. Additionally, methylene blue may scavenge NO directly and has antioxidant properties that could contribute to its beneficial effects in septic shock [27].

Evidence for methylene blue in septic shock

Investigation into methylene blue's potential role in septic shock began in the late 1990s and early 2000s, with Preiser et al. conducting one of the first pilot studies in 1995, evaluating methylene blue in five patients with septic shock and reporting temporary increases in blood pressure with minimal effects on cardiac performance [28]. The first randomized controlled pilot study was published in 2001 by Kirov et al., who compared methylene blue (n = 10) to isotonic saline (n = 10) as adjunctive therapy in septic shock patients, with the intervention group receiving a 2 mg/kg bolus followed by an infusion at stepwise increasing rates, and observed that methylene blue reduced concurrent adrenergic support requirements without affecting oxygen delivery or other hemodynamic parameters [29]. Memis et al. conducted a randomized trial in 2002 focusing on cytokine levels during septic shock, administering methylene blue at 0.5 mg/kg/hour, and while they found no significant effect on cytokine levels or mortality, they did observe increased mean arterial pressure in the treatment group [30].

Recent years have seen renewed interest in methylene blue for septic shock, with several notable studies. Ibarra-Estrada et al. conducted a single-center randomized controlled trial (RCT) in 2023 enrolling patients with septic shock requiring norepinephrine, where the treatment group received 500 mg of methylene blue over six hours daily for three consecutive days, while controls received equivalent volumes of 0.9% saline. This relatively high-dose protocol resulted in statistically significant earlier discontinuation of vasopressors (median three vs. four days), one additional vasopressor-free day at day 28, and shorter intensive care unit (ICU) and hospital lengths of stay, though no difference in mortality or serious adverse events was observed [31].

Shaker et al. compared two different dosing strategies in 2025: 1 mg/kg vs. 4 mg/kg bolus, both followed by 0.25 mg/kg/hour infusion for 72 hours. Both dosing regimens significantly reduced norepinephrine requirements compared to saline controls, with no difference between the two active treatment groups; however, after logistic regression analysis, the 4 mg/kg group demonstrated a reduced mortality risk (hazard ratio: 0.29, 95% CI: 0.09-0.90) [32].

Kannan and Syeda published a case series in 2023 of septic shock patients already receiving maximum doses of norepinephrine (≥0.2 μg/kg/min) and vasopressin (0.04 IU/min), administering methylene blue as a 3 mg/kg bolus followed by 0.5 mg/kg/min for 48 hours. All patients demonstrated reduced vasopressor requirements and lactate levels, and interestingly, they observed reductions in inflammatory cytokine levels, suggesting additional anti-inflammatory effects beyond vasopressor-sparing action [33].

Kuri et al. conducted a direct head-to-head comparison between methylene blue and vasopressin as second-line agents in septic shock in 2025, where patients requiring norepinephrine ≥ 0.2 μg/kg/min received either methylene blue (1 mg/kg bolus followed by 0.5 mg/kg infusion over six hours) or vasopressin (0.04 units/min for six hours). While there was no significant difference in norepinephrine dose between groups at 0 and six hours, the vasopressin group had significantly lower norepinephrine requirements between 12 and 24 hours, and additionally, lactate levels and SOFA scores at 24 hours were significantly lower in the vasopressin group [34].

Two recent meta-analyses have evaluated the efficacy of methylene blue in critical illness. Fernando et al. conducted a systematic review and meta-analysis in 2024 of six RCTs in septic shock, finding low-certainty evidence suggesting that methylene blue might reduce the duration of vasopressor support, hospital length of stay, and possibly short-term mortality, with no increase in adverse events noted [35]. Pruna et al. performed a broader meta-analysis in 2024 including 11 studies (both randomized and non-randomized) across various critical care scenarios, including septic shock and post-cardiac surgery vasoplegic syndrome, reporting that methylene blue use was associated with significantly lower mortality in both septic shock and cardiac surgery subgroups, as well as reduced ICU and hospital length of stay, and hemodynamically, methylene blue patients had higher mean arterial pressure and systemic vascular resistance without changes in cardiac output [36].

Practical considerations for clinical use

The optimal dosing of methylene blue in septic shock remains undefined, with various regimens reported in the literature, including bolus dosing ranging from 1 to 4 mg/kg typically administered over 20-60 minutes, continuous infusion ranging from 0.25 to 1.0 mg/kg/hour with durations from six to 72 hours, and a combined approach of initial bolus followed by continuous infusion. The Shaker et al. study suggests that higher bolus doses (4 mg/kg vs. 1 mg/kg) might be associated with mortality benefit, though both dosing regimens provided similar vasopressor-sparing effects [32]. The Ibarra-Estrada protocol using 500 mg daily (approximately 5-7 mg/kg for an average adult) for three consecutive days showed promising results but differs substantially from earlier studies using lower doses [31].

The optimal timing for methylene blue administration remains unclear, as most studies have evaluated it as an adjunctive therapy in established septic shock, after the initiation of standard vasopressors, and whether earlier administration could provide additional benefits by preventing the progression to refractory shock requires further investigation. The Kuri et al. study comparing methylene blue to vasopressin suggests that the latter may be more effective as a second-line agent, particularly beyond the immediate six-hour window [34], raising questions about whether methylene blue might be more suitable as a third-line agent or for specific patient subgroups.

Important pharmaceutical considerations include dilution, as methylene blue is a hypotonic solution and should not be diluted in normal saline due to risk of precipitation, with recommended diluents including 5% dextrose or sterile water [37]; compatibility concerns, as methylene blue is incompatible with sodium bicarbonate and compatibility data with other commonly used ICU medications are limited [38]; and administration rate, as rapid administration (<15-20 minutes) has been associated with hypotension, possibly due to transient inhibition of eNOS before the more sustained inhibition of iNOS becomes apparent [39].

Methylene blue is generally well-tolerated when administered at recommended doses (1-4 mg/kg), but potential adverse effects include blue-green discoloration of urine, skin, and mucous membranes (common but harmless) [40]; hemolytic anemia, particularly in patients with glucose-6-phosphate dehydrogenase (G6PD) deficiency, as methylene blue can induce oxidative stress in red blood cells [41]; serotonin syndrome when administered to patients taking serotonergic medications (selective serotonin reuptake inhibitors (SSRIs), serotonin-norepinephrine reuptake inhibitors (SNRIs), and monoamine oxidase inhibitors (MAOIs)), due to methylene blue's action as a monoamine oxidase inhibitor [42]; interference with pulse oximetry, potentially causing falsely decreased SpO_2_ readings due to absorbance spectra overlapping with oxyhemoglobin [43]; and cardiac arrhythmias reported with rapid administration or excessive doses [44].

Absolute contraindications include confirmed G6PD deficiency, concurrent use of serotonergic medications, and hypersensitivity to methylene blue or similar thiazine dyes, while relative contraindications include severe renal impairment (CrCl < 30 mL/min), severe hepatic dysfunction, and pregnancy (safety not established).

Future directions and research gaps

Despite growing interest in methylene blue for septic shock, several important questions remain unanswered. These include identifying optimal patient selection through specific biomarkers or clinical characteristics that predict favorable response to methylene blue therapy; determining standardized dosing protocols for optimal bolus doses, infusion rates, and duration of therapy; evaluating the timing of intervention and whether early administration provides additional benefits compared to rescue therapy in refractory shock; investigating potential synergistic effects when combined with other vasoactive agents, particularly non-catecholamine vasopressors like vasopressin [9] or angiotensin II [10]; further exploring potential immunomodulatory and anti-inflammatory properties observed in some studies; and examining long-term outcomes, as most studies have focused on short-term endpoints like vasopressor requirements and ICU length of stay, with limited data on long-term mortality and functional outcomes.

Ongoing and future research should focus on large, multicenter RCTs with standardized protocols and comprehensive outcome measures, while translational research investigating the molecular and cellular effects of methylene blue in sepsis could provide insights into optimizing therapeutic strategies.

## Conclusions

Methylene blue represents a mechanistically rational adjunctive therapy for septic shock, targeting the NO-cyclic GMP pathway implicated in vasoplegic shock. The growing body of evidence suggests potential benefits including reduced vasopressor requirements, shorter ICU length of stay, and possibly improved survival in specific patient populations. However, current evidence does not support methylene blue as a replacement for established second-line agents like vasopressin, and it may be best positioned as a third-line agent in refractory septic shock or as a temporizing measure while more definitive interventions are arranged. Until more robust evidence becomes available through further high-quality clinical trials, clinicians should carefully weigh potential benefits against risks, particularly in vulnerable populations, with close monitoring of hemodynamic parameters, tissue perfusion markers, and potential adverse effects during administration.
